# Perceptions, attitudes and beliefs towards soya among healthy Asian women participating in a soya randomised controlled trial

**DOI:** 10.1017/jns.2023.48

**Published:** 2023-07-05

**Authors:** Beverley Yap, Nadia Rajaram, Weang Kee Ho, Geok Lin Khor, Soo Hwang Teo

**Affiliations:** 1 Cancer Research Malaysia, Subang Jaya, Selangor 47500, Malaysia; 2 School of Mathematical Sciences, Faculty of Science and Engineering, University of Nottingham Malaysia, Semenyih, Selangor 43500, Malaysia; 3 Department of Nutrition and Dietetics, Faculty of Medicine and Health Sciences, Universiti Putra Malaysia, Serdang 43400, Malaysia; 4 Faculty of Medicine, University Malaya Cancer Research Institute, University of Malaya, Kuala Lumpur 50603, Malaysia

**Keywords:** Asian, COM-B model, Perception, Post-menopausal women, Soya, Theoretical Domains Framework

## Abstract

The soya–breast cancer risk relationship remains controversial in Asia due to limited and inconsistent research findings and is exacerbated by difficulties in recruiting and retaining participants in intervention trials. Understanding public perceptions towards soya is important for designing effective intervention trials. Here, we administered a close-ended, quantitative survey to healthy, peri- and post-menopausal Asian women in the Malaysian Soy and Mammographic Density (MiSo) Study to assess perception towards soya and explore motivators and barriers that affect study adherence using the Capability, Opportunity, Motivation and Belief (COM-B) Model and Theoretical Domains Framework (TDF). Of 118 participants, the majority reported the belief that soya promotes good health (Supplement = 85⋅7 %, Diet = 90⋅0 %, Control = 87⋅9 %). Most participants reported obtaining information about soya from the internet (Supplement = 61⋅0 %, Diet = 55⋅3 %, Control = 35⋅9 %), while health professionals were least reported (Supplement = 9⋅8 %, Diet = 7⋅9 %, Control = 5⋅1 %). Stratified analyses by study completion and adherence status yielded comparable findings. By the end of the study, dietary arm participants reported a strong belief that soya has no impact on their health (Supplement = 7⋅1 % *v.* Diet = 20⋅0 % *v.* Control = 0⋅0 %, *P* = 0⋅012). Motivation and opportunity strongly facilitated soya consumption, while psychological capability was the most common barrier to consumption though less evident among dietary arm participants. While most Asian women have a positive perception towards soya, theory-based intervention trials are warranted to understand the perception–study adherence relationship and to accurately inform the public of the health effects of soya.

## Introduction

Soya foods are known to be an abundant source of dietary isoflavones and are commonly a staple in Asian diets^(1)^. Since the first report of lower breast cancer risk among Chinese women with high soya intake^(2)^, the soya–breast cancer risk relationship has been extensively investigated. Research has suggested that dietary soya intake may have a protective effect against various chronic diseases and cancers^(1,3–8)^, but randomised controlled trials have reported little to no association between soya intake and the risk of breast cancer^(9–13)^.

Unsurprisingly, public perception of the association between soya and cancer risk remains controversial^(4,7,14)^. There has been limited research on the population's perception of soya and how it affects their consumption patterns. Studies have described the attitudes, beliefs, motivators and barriers to consuming soya within Western populations^(15–21)^. These studies reported that Western populations had a positive perception towards soya and commonly consumed soya to engage in a healthy diet while the major barriers to consumption were disliking the taste of soya and the lack of knowledge in preparing soya meals^(15–21)^. Although soya foods have been extensively explored for its health effects as a source of protein^(1,22,23)^, there are limited reports describing the motivators and barriers to soya consumption among women living in Asia, nor how this could affect their willingness to consume soya, or to participate effectively in intervention studies that investigate the potential health benefits of soya.

The Capability, Opportunity, Motivation and Behaviour (COM-B) model is a behavioural system that explores how capability, opportunity and motivation interact to produce behaviour^(24)^. The Theoretical Domains Framework (TDF), on the other hand, is a holistic theoretical framework that characterises the determinants of human behaviour and is typically utilised in the designing of new interventions^(25–28)^. The COM-B model and TDF have been shown to work synergistically, and have been used in several studies to characterise the motivators and barriers to nutritional/dietary behaviours^(29,30)^.

In the present study, we describe the perception, attitudes and beliefs towards soya consumption in a cohort of healthy, peri- and post-menopausal Asian women participating in a soya dietary intervention trial and explore how these factors may affect the completion of the study and adherence to the intervention. Furthermore, using the COM-B model and TDF, we describe the motivators and barriers to regular soya consumption.

## Materials and methods

### Study design

**Fig. 1.**
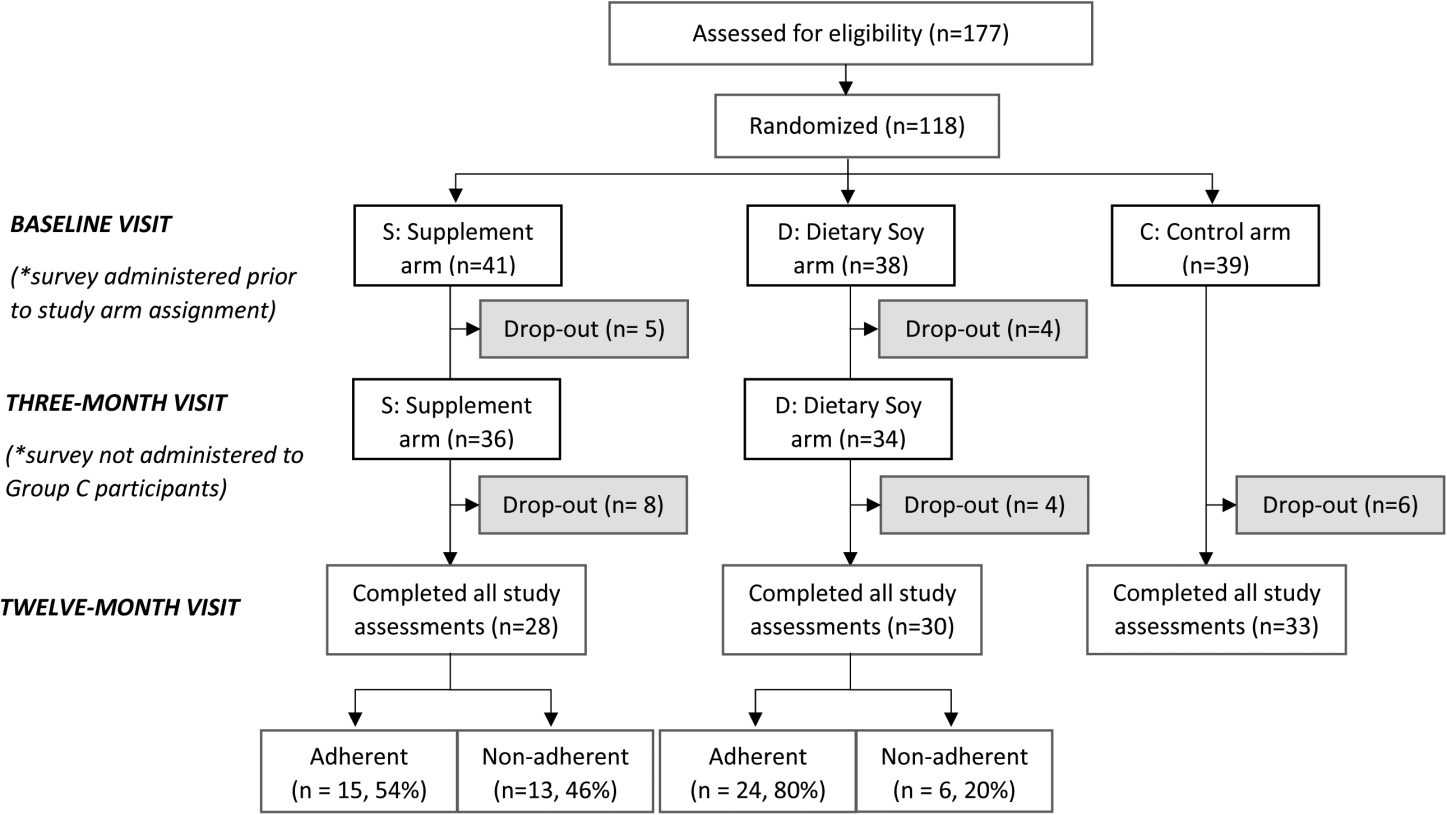
CONSORT flow diagram from enrolment to completion of the MiSo Study. Drop-out participants include those who were lost to follow-up or discontinued the intervention. Women were considered ‘adherent’ if they had an intervention compliance of more than 80 %. ‘Non-adherent’ participants had an intervention compliance of less than 80 %.

The Malaysian Soy and Mammographic Density (MiSo) Study^(31)^ is a three-arm, open-labelled, randomised controlled clinical trial that aimed to investigate the impact of soya isoflavone intake for 1 year on mammographic density as a biomarker for breast cancer risk among peri- and post-menopausal Asian women. Participants were recruited from an existing database of women who previously participated in the Malaysian Mammography (MyMammo) screening programme, an opportunistic screening programme that offered subsidised mammograms for women who consented to participate^(32)^. Participants were also recruited through social media, newspaper advertisements and brochures placed in clinics at a tertiary private hospital (Subang Jaya Medical Centre). Eligible women were randomised to receive either 100 mg/d of isoflavones through supplements, 50 mg/d of isoflavones through a high soya diet, or were a negative control (i.e. no changes to diet) (Fig. 1).

This study was conducted according to the guidelines laid down in the Declaration of Helsinki and all procedures involving research study participants were approved by the Ramsay Sime Darby Healthcare Independent Ethics Committee (Reference #: 201805.1, 12 July 2018) and the University Malaya Medical Centre Medical Research (Reference #: 202043-8441, 22 April 2020). The study is also registered on clinicaltrials.gov (Reference #: NCT03686098). Written informed consent was obtained from all participants of the study.

### Study population

A total of 177 women were assessed from December 2018 to October 2020. Women were excluded if they were previously diagnosed with any breast, cardiovascular, inflammatory or gastrointestinal diseases, or if they had gout, diabetes, hypothyroidism, soya allergy or were on hormone replacement therapies. Women who had a high soya intake (at least one serving of soya per day) or had menstruation less than 3 months from the consent date or a mammogram in the past 12 months were also excluded from the study. After exclusions, 118 participants were randomised, using a computer-generated randomisation list generated by the study manager, to either the negative control arm, soya isoflavone supplement arm or dietary soya arm by the study coordinator. A stratified, block randomisation approach was used to account for potential differences in distribution by ethnicity and menopausal status. The randomisation list was filed in the Trial Master File and concealed until interventions were assigned. Due to the nature of the study treatments, all participants, the study manager and the study coordinator were not blinded to treatment allocation.

### Data collection

A close-ended quantitative survey consisting of multiple-choice questions about perceptions, attitudes, beliefs towards soya, and motivators and barriers to regular soya consumption was designed from qualitative data collected from a feasibility study. In the feasibility study, ten Asian women with the same selection criteria as the MiSo Study were asked to consume 100 mg of soya for 2 months and were then invited for a semi-structured interview at the end of the first month to explore the motivators and barriers to participation in a dietary soya intervention study. The themes identified were mapped to the COM-B model and TDF (Supplementary Table S1). Within the COM-B model, capability relates to the physical or psychological skills and ability of an individual to carry out a behaviour. Opportunity relates to the external physical and social influences that drive behaviour change. Finally, motivation refers to the intrinsic processes that influence decision-making and behaviours, such as reflective motivation (i.e. reflective processes such as making plans) and automatic motivation (i.e. desires, impulses and inhibitions).

The survey was administered to all participants (*n* 118) of the MiSo Study at baseline and 12-month visits at a tertiary private hospital (Subang Jaya Medical Centre). In addition, the survey was administered to participants in the supplement and dietary intervention arms at their 3-month visit.

### Study outcomes

The primary outcome of this perception study was to understand the perceptions, motivators and barriers to soya consumption among healthy, post-menopausal Asian women participating in a soya intervention trial through the administration of the aforementioned quantitative survey at baseline, 3-month and end-of-study visits.

### Data analysis

All participants were analysed by study arm, where participants in the supplement arm are referred to as Group S, the dietary arm as Group D and the control arm as Group C. Only participants within Group S and Group D were included in the analysis by study completion status: participants who completed all study assessments as per protocol were defined as ‘complete’ while those who discontinued the intervention or were lost to follow-up were categorised as ‘drop-out’. Women who completed the study and maintained an intervention compliance of 80 % or more were defined as ‘adherent’ while those below 80 % were defined as ‘non-adherent’.

The age of participants at enrolment and age at menarche (both in years) were described using mean and standard deviation. Student's *t* test was used to test for differences in age and age at menarche by retention status. All remaining survey data such as socio-demographic factors, menopausal status, medical history and health beliefs were described in frequencies. Motivators and barriers to regular soya consumption were deductively mapped to the TDF and COM-B domains^(24,26)^ and described in frequencies. A Fisher's Exact test was used to test for differences by retention status and McNemar's test was used to calculate perception change over time.

All hypotheses were two-sided, and a *P*-value of <0⋅05 was considered statistically significant. The analysis was done using the RStudio statistical environment software (version 1.3.1093).

## Results

### Participant's characteristics

In this year-long soya intervention trial, 118 healthy, Asian peri- and post-menopausal women were enrolled and randomised into one of the three study arms (Fig. 1). Among the participants assigned to an intervention (Group S or Group D), 58 (73⋅4 %) women completed the study as per the protocol and 21 (26⋅6 %) participants discontinued the intervention or were lost to follow-up (Table 1). Out of those who completed the study, 39 (67⋅2 %) women adhered to the intervention and 19 (32⋅8 %) were non- or partially adherent.

**Table 1.** Patient characteristics at baseline visit for overall cohort, by study arm, study completion status and intervention adherence status

Participant characteristic	All (*n* 118)	Study arm (%)				SCS (%)			Intervention adherence (%)		
		S (*n* 41)	D (*n* 38)	C (*n* 39)	*P*	Complete (*n* 58)	Drop-out (*n* 21)	*P*	Adh (*n* 39)	Non-adh (*n* 19)	*P*
Age in years, mean (sd)	56⋅6 (4⋅5)	56⋅8 (4⋅4)	56⋅8 (4⋅2)	56⋅1 (4⋅9)	0⋅69	56⋅8 (4⋅3)	57⋅4 (4⋅3)	0⋅794	56⋅6 (4⋅3)	57⋅1 (4⋅4)	0⋅721
Ethnicity, *n* (%)											
Chinese	92 (78⋅0)	31 (75⋅6)	31 (81⋅6)	30 (76⋅9)	0⋅967	46 (79⋅3)	16 (76⋅2)	0⋅906	32 (82⋅1)	14 (73⋅7)	0⋅784
Indian	16 (13⋅6)	6 (14⋅6)	4 (10⋅5)	6 (15⋅4)		7 (12⋅1)	3 (14⋅3)		5 (12⋅8)	2 (10⋅5)	
Malay	10 (8⋅5)	4 (9⋅8)	3 (7⋅9)	3 (7⋅7)		5 (8⋅6)	2 (9⋅5)		2 (5⋅1)	3 (15⋅8)	
Education, *n* (%)											
Certification/Tertiary	80 (67⋅8)	26 (63⋅4)	23 (60⋅5)	31 (79⋅5)	0⋅273	36 (62⋅1)	13 (61⋅9)	0⋅788	22 (56⋅4)	14 (73⋅7)	0⋅201
Primary/Secondary	34 (28⋅8)	13 (31⋅7)	13 (34⋅2)	8 (20⋅5)		20 (34⋅5)	6 (28⋅6)		16 (41⋅0)	4 (21⋅1)	
Monthly household income, *n* (%)											
B40 (<RM 5000)	42 (35⋅6)	14 (34⋅1)	14 (36⋅8)	14 (35⋅9)	0⋅991	22 (37⋅9)	6 (28⋅6)	0⋅841	17 (43⋅6)	5 (26⋅3)	0⋅634
M40 (RM 5000–10 000)	38 (32⋅2)	14 (34⋅1)	11 (28⋅9)	13 (33⋅3)		18 (31⋅0)	7 (33⋅3)		10 (25⋅6)	8 (42⋅1)	
T40 (RM 10 000–RM 50 000)	33 (28⋅0)	11 (26⋅8)	10 (26⋅3)	12 (30⋅8)		15 (25⋅9)	6 (28⋅6)		10 (25⋅6)	5 (26⋅3)	
Age at menarche, median (sd)	12⋅6 (1⋅3)	12⋅5 (1⋅1)	12⋅3 (1⋅3)	12⋅8 (1⋅5)	0⋅241	12⋅4 (1⋅1)	12⋅6 (1⋅5)	0⋅452	12⋅3 (1⋅1)	12⋅4 (1⋅2)	0⋅686
Menopausal status, *n* (%)											
Post-menopausal	104 (88⋅1)	35 (85⋅4)	34 (89⋅5)	35 (89⋅7)	0⋅824	52 (89⋅7)	17 (81⋅0)	0⋅443	36 (92⋅3)	16 (84⋅2)	0⋅596
Peri-menopausal	14 (11⋅9)	6 (14⋅6)	4 (10⋅5)	4 (10⋅3)		6 (10⋅3)	4 (19⋅0)		3 (7⋅7)	3 (15⋅8)	
First FH of any cancer, *n* (%)	52 (44⋅1)	20 (48⋅8)	16 (42⋅1)	16 (41⋅0)	0⋅694	28 (48⋅3)	8 (38⋅1)	0⋅449	20 (51⋅3)	8 (42⋅1)	0⋅537
First FH of breast cancer, *n* (%)	14 (11⋅9)	5 (12⋅2)	5 (13⋅2)	4 (10⋅3)	0⋅94	7 (12⋅1)	3 (14⋅3)	0⋅999	5 (12⋅8)	2 (10⋅5)	0⋅909
Years since last mammogram, *n* (%)											
Within 2 years	36 (30⋅5)	13 (31⋅7)	13 (34⋅2)	10 (25⋅6)	0⋅564	21 (36⋅2)	5 (23⋅8)	0⋅516	16 (41⋅0)	5 (26⋅3)	0⋅304
More than 2 years	79 (66⋅9)	27 (65⋅9)	23 (60⋅5)	29 (74⋅4)		35 (60⋅3)	15 (71⋅4)		21 (53⋅8)	14 (73⋅7)	
Never	3 (2⋅5)	1 (2⋅4)	2 (5⋅3)	0 (0⋅0)		2 (3⋅4)	1 (4⋅8)		2 (5⋅1)	0 (0⋅0)	

SCS, study completion status; S, supplement arm; D; dietary arm; C, control arm; *P*, *P*-value calculated using a Fisher's Exact test; Adh, adherence; Non-adh, non-adherence; sd, standard deviation; RM, Ringgit Malaysia; FH, family history.

As seen in Fig. 1, ~23 % of participants dropped out of the study. Most women were lost to follow-up due to the onset of adverse events, followed by a lack of interest in the study and the COVID-19 pandemic^(31)^.

Table 1 shows the demographics of the whole cohort by their study arm, study completion status and intervention adherence status at the baseline. Women were between 45 and 65 years of age, with an average age of 57 years. Most participants were post-menopausal (88⋅1 %) and have had a mammogram more than 2 years since the date of enrolment (66⋅9 %). Women in this study were mostly Chinese (78⋅0 %), followed by Indians (13⋅6 %) and Malays (8⋅5 %). Participants were likely to have completed some tertiary education (67⋅8 %) and have at least one first-degree relative with cancer (44⋅1 %). There were no significant differences in socio-demographic, reproductive or family history of cancer by study arm, study completion status and intervention adherence status (Table 1).

### Availability and utilisation

The analysis of availability and utilisation of soya products was investigated using data collected from participants at their baseline visits. The majority of participants reported purchasing soya products from grocery stores and this did not differ by study arm (S = 78⋅0 %, D = 76⋅3 %, C = 71⋅8, *P* = 0⋅831) (Supplementary Table S2). Compared to participants in the supplement and dietary arm, women in the control arm were more likely to report purchasing soya products from the wet market (S *v.* D *v.* C: 24⋅4 % *v.* 39⋅5 % *v.* 53⋅8 %, *P* = 0⋅027) (Table 2). Irrespective of study arms, most women reported seeking information about soya from the internet (S = 61⋅0 %, D = 55⋅3 %, C = 56⋅4 %, *P* = 0⋅893), and were least likely to seek information from health professionals (S = 9⋅8 %, D = 7⋅9 %, C = 5⋅1 %, *P* = 0⋅835) such as doctors, nurses or nutritionists. The observation from the analyses by completion and adherence status were similar, in which irrespective of study completion or adherence to the intervention, most women reported purchasing soya products from grocery stores, are most likely to seek information regarding soya on the internet and are least likely to seek information from health professionals. Interestingly, compared to women who were adherent, women who were non-adherent were more likely to obtain information about soya from public talks (2⋅6 % *v.* 21⋅1 %, *P* = 0⋅036) (Supplementary Table S3).

**Table 2.** Perception of health effects of soya, motivators and barriers to the regular consumption of soya by study arm at baseline, end-of-study and change over time

Perception statement	Baseline				End-of-study				Change over time					
	S	D	C	*P*^‡^	S	D	C	*P*^‡^	S	*P*^‡^	D	*P*^‡^	C	*P*^‡^
Positive statement														
Promotes good health	34 (82⋅9)	31 (81⋅6)	30 (76⋅9)	0⋅839	24 (85⋅7)	27 (90⋅0)	29 (87⋅9)	0⋅922	2⋅80 %	0⋅999	8⋅40 %	0⋅999	11⋅00 %	0⋅999
Promotes healthy pregnancy	14 (34⋅1)	15 (39⋅5)	16 (41⋅0)	0⋅792	4 (14⋅3)	0 (0⋅0)	14 (42⋅4)	0⋅055	−19⋅80 %	0⋅041	−39⋅50 %	0⋅789	1⋅40 %	0⋅999
Helps reduce menopause symptoms	10 (24⋅4)	10 (26⋅3)	10 (25⋅6)	0⋅999	7 (25⋅0)	13(43⋅3)	9 (27⋅3)	0⋅279	0⋅60 %	0⋅999	17⋅00 %	0⋅077	1⋅70 %	0⋅999
May reduce risk of BC	7 (17⋅1)	11 (28⋅9)	13 (33⋅3)	0⋅239	9 (32⋅1)	17 (56⋅7)	14 (42⋅4)	0⋅179	15⋅00 %	0⋅724	27⋅80 %	0⋅027*	9⋅10 %	0⋅724
Neutral statement														
Has no impact on health	2 (4⋅9)	5 (13⋅2)	4 (10⋅3)	0⋅415	2 (7⋅1)	6 (20⋅0)	0 (0⋅0)	0⋅012*	2⋅20 %	NA	6⋅80 %	0⋅999	−10⋅30 %	NA
I have heard other things about soya	5 (12⋅2)	3 (7⋅9)	5 (12⋅8)	0⋅813	1 (3⋅6)	0 (0⋅0)	3 (9⋅1)	0⋅260	−8⋅60 %	0⋅248	−7⋅90 %	NA	−3⋅70 %	0⋅999
I have not heard anything about soya and health	2 (4⋅9)	1 (2⋅6)	4 (10⋅3)	0⋅394	2 (7⋅1)	0 (0⋅0)	0 (0⋅0)	0⋅092	2⋅20 %	0⋅999	−2⋅60 %	NA	−10⋅30 %	NA
Negative statement														
Harmful to health	4 (9⋅8)	6 (15⋅8)	1 (2⋅6)	0⋅128	2 (7⋅1)	0 (0⋅0)	1 (3⋅0)	0⋅402	−2⋅70 %	0⋅617	−15⋅80 %	NA	0⋅40 %	NA
Worsens gout	13 (31⋅7)	11 (28⋅9)	8 (20⋅5)	0⋅517	7 (25⋅0)	11 (36⋅7)	12 (36⋅4)	0⋅524	−6⋅70 %	0⋅131	7⋅80 %	0⋅289	15⋅90 %	0⋅228
Worsens thyroid issues	1 (2⋅4)	0 (0⋅0)	1 (2⋅6)	0⋅999	2 (7⋅1)	1 (3⋅3)	2 (6⋅1)	0⋅862	4⋅70 %	0⋅999	3⋅30 %	NA	3⋅50 %	0⋅999
May cause cancer, including BC	2 (4⋅9)	5 (13⋅2)	7 (17⋅9)	0⋅178	4 (14⋅3)	4 (13⋅3)	4 (12⋅1)	0⋅999	9⋅40 %	0⋅617	0⋅10 %	0⋅999	−5⋅80 %	0⋅450
May trigger cancer, if there is cancer in the family	2 (4⋅9)	1 (2⋅6)	2 (5⋅1)	0⋅999	1 (3⋅6)	1 (3⋅3)	0 (0⋅0)	0⋅533	−1⋅30 %	0⋅999	0⋅70 %	0⋅999	−5⋅10 %	NA
Motivators														
Automatic motivation	33 (80⋅5)	28 (73⋅7)	23 (59⋅0)	0⋅110	17 (60⋅7)	25 (83⋅3)	23 (69⋅7)	0⋅155	−19⋅80 %	0⋅114	9⋅60 %	0⋅371	10⋅70 %	0⋅289
Ref. Mot. (belief about consequences)	30 (73⋅2)	22 (57⋅9)	17 (43⋅6)	0⋅028*	15 (53⋅6)	21 (70⋅0)	20 (60⋅6)	0⋅453	−19⋅60 %	0⋅114	12⋅10 %	0⋅343	17⋅00 %	0⋅149
Ref. Mot. (intentions)	18 (43⋅9)	14 (36⋅8)	14 (35⋅9)	0⋅749	8 (28⋅6)	21 (70⋅0)	17 (51⋅5)	0⋅008*	−15⋅30 %	0⋅343	33⋅20 %	0⋅039*	15⋅60 %	0⋅182
Social opportunity	24 (58⋅5)	30 (78⋅9)	23 (59⋅0)	0⋅100	15 (53⋅6)	24 (80⋅0)	23 (69⋅7)	0⋅102	−4⋅90 %	0⋅724	1⋅10 %	0⋅999	10⋅70 %	0⋅773
Physical opportunity	26 (63⋅4)	23 (60⋅5)	18 (46⋅2)	0⋅265	18 (64⋅3)	24 (80⋅0)	24 (72⋅7)	0⋅445	0⋅90 %	0⋅999	19⋅50 %	0⋅114	26⋅50 %	0⋅146
Other reasons	2 (4⋅9)	2 (5⋅3)	3 (7⋅7)	0⋅895	1 (3⋅6)	1 (3⋅3)	4 (12⋅1)	0⋅363	−1⋅30 %	0⋅999	−2⋅00 %	0⋅999	4⋅40 %	0⋅480
Barriers														
Psychological capability	14 (34⋅1)	11 (28⋅9)	19 (48⋅7)	0⋅191	7 (25⋅0)	1 (3⋅3)	9 (27⋅3)	0⋅018*	−9⋅10 %	0⋅289	−25⋅60 %	0⋅027*	−21⋅40 %	0⋅027*
Social opportunity	6 (14⋅6)	3 (7⋅9)	9 (23⋅1)	0⋅179	4 (14⋅3)	1 (3⋅3)	3 (9⋅1)	0⋅377	−0⋅30 %	0⋅683	−4⋅60 %	0⋅999	−14⋅00 %	0⋅450
Reflective motivation	4 (9⋅8)	4 (10⋅5)	7 (17⋅9)	0⋅577	3 (10⋅7)	2 (6⋅7)	5 (15⋅2)	0⋅589	0⋅90 %	0⋅999	−3⋅80 %	0⋅999	−2⋅70 %	0⋅999
Automatic motivation	0 (0⋅0)	3 (7⋅9)	3 (7⋅7)	0⋅188	0 (0⋅0)	1 (3⋅3)	1 (3⋅0)	0⋅999	0⋅00 %	NA	−4⋅60 %	0⋅480	−4⋅70 %	0⋅617
Physical opportunity	1 (2⋅4)	1 (2⋅6)	2 (5⋅1)	0⋅842	0 (0⋅0)	1 (3⋅3)	1 (3⋅0)	0⋅999	−2⋅40 %	NA	0⋅70 %	0⋅999	−2⋅10 %	0⋅999
Other reasons	2 (4⋅9)	1 (2⋅6)	5 (12⋅8)	0⋅216	2 (7⋅1)	2 (6⋅7)	4 (12⋅1)	0⋅727	2⋅20 %	0⋅999	4⋅10 %	0⋅999	−0⋅70 %	0⋅999

S, supplement arm; D; dietary arm; C, control arm; BC, breast cancer; Ref. Mot., reflective motivation.

† *P*-value calculated for study arms S, D and C using a Fisher's Exact test.

‡ *P*-value calculated to for perception change over time with McNemar's test.

* *P* < 0⋅05.

### Health beliefs about soya

There were no differences by study arms at the baseline (Table 2). At the 3-month visit, compared to Group S participants, participants in Group D were more likely to believe that soya may cause cancer, including breast cancer (0⋅0 % *v.* 14⋅7 %, *P* = 0⋅023) (Supplementary Table S4). This comparison was no longer significant at the end of the study (Table 2). At the end of the study, irrespective of study arms, a majority of participants reported a strong belief that soya promotes good health (S = 85⋅7 %, D = 90⋅0 %, C = 87⋅9 %, *P* = 0⋅922), reduces menopausal symptoms (S = 25⋅0 %, D = 43⋅3 %, C = 27⋅3 %, *P* = 0⋅279) and may reduce risk of breast cancer (S = 32⋅1 %, D = 56⋅7 %, C = 42⋅4 %, *P* = 0⋅179) (Table 2). Some participants had a negative perception towards soya, the most commonly reported belief being that it worsens gout (S = 25⋅0 %, D = 36⋅7 %, C = 36⋅4 %, *P* = 0⋅524) (Table 2). Interestingly, compared to participants in Group S and Group C, participants in Group D were more likely to believe that soya has no impact on health at the end of the study (S *v.* D *v.* C: 7⋅1 % *v.* 20⋅0 % *v.* C = 0⋅0 %, *P* = 0⋅012) and also had increased belief over time that soya may reduce their risk of breast cancer (*P* = 0⋅027; Table 2). While our analysis only has approximately 45 % power to detect a difference in perception across the three study arms at the end of the study, it is approximately 70 % powered to detect this change in perception over time (Supplementary Table S5). There were no differences in study completion and intervention adherence status (Supplementary Table S3). Additionally, there were no differences by ethnicity observed (data not shown).

### Motivators and barriers to regular soya consumption within the COM-B model and TDF

Within the motivation domain of the COM-B model, automatic motivation was one of the most commonly reported reasons why women consumed soya regularly at their 12-month visit, and this did not differ by study arms (S = 60⋅7 %, D = 83⋅3 %, C = 69⋅7 %, *P* = 0⋅155) (Table 2). This is further described by the TDF concept of reinforcement and had largely to do with participant's degree of enjoyment for soya products (Supplementary Table S1). A similar observation was found in the analyses by study completion and adherence status, wherein automatic motivation was one of the most commonly reported reasons for consumption at the final visit of the study (Supplementary Table S3).

Reflective motivation was also a commonly reported motivator, and maps to belief about consequences and intentions within the TDF. Such motivators include the belief that soya is good for health and to include soya in a regular diet (Supplementary Table S1). At baseline, compared to Groups D and C, participants in Group S were more likely to report belief about consequences as a motivator to consumption (S *v.* D *v.* C: 73⋅2 % *v.* 57⋅9 % *v.* 43⋅6 %, *P* = 0⋅028; Table 2), though this was no longer observed at the final study visit. Conversely, at the end of the study, participants in Group D were more likely to report intentions as a motivator to consumption, while Group S participants were least likely to report this as a motivator (S *v.* D *v.* C: 28⋅6 % *v.* 70⋅0 % *v.* 60⋅6 %, *P* = 0⋅008). We also see a significant increase in intentions as a motivator to consumption over time among the participants in Group D (36⋅8 % *v.* 70⋅0 %, *P* = 0⋅039). There were no differences between study completion and adherence status with reflective motivation as a motivator, but differences were observed for reflective motivation as a barrier (Supplementary Table S3). At the 3-month visit, ‘drop-out’ participants were more likely to report reflective motivation as a barrier to consumption (Complete *v.* Drop-out: 3⋅5 % *v.* 38⋅5 %, *P* = 0⋅002) (Supplementary Table S3). Additionally, reflective motivation was more commonly reported as a barrier among non-adherent participants (2⋅6 % *v.* 21⋅1 %, *P* = 0⋅036; Supplementary Table S3). Such barriers include the belief that soya is bad for health or that it causes bloating or weight gain (data not shown). All significant results were more than 80 % powered to detect a difference (Supplementary Table S5).

At the final study visit, participants also reported social opportunity as an important motivator to the regular consumption of soya, and this was observed across all three study arms (S = 53⋅6 %, D = 80⋅0 %, C = 69⋅7 %, *P* = 0⋅102) (Table 2). These social influences include their family, friends, doctors, the media and culture, with family being the most influencing factor (data not shown). Apart from that, motivators relating to physical opportunities, such as the affordability and accessibility of soya products, were also commonly reported (S = 64⋅3 %, D = 80⋅0 %, C = 72⋅7 %, *P* = 0⋅445) (Table 2). There were no differences in study completion and intervention adherence status.

While motivation and opportunity were largely reported as motivators to regular consumption, the psychological capability was the largest barrier to regular consumption, though this was more commonly reported by participants in Group S and Group C, and significantly less likely for Group D participants at the end of the study (S *v.* D *v.* C: 25⋅0 % *v.* 3⋅3 % *v.* 27⋅3 %, *P* = 0⋅018) (Table 2). Our results also show that psychological capacity as a barrier changed over time among participants in the dietary and control arm (S *v.* D *v.* C *P* = 0⋅289 *v.* 0⋅027 *v.* 0⋅027). These appear to be driven by the knowledge domain of the TDF and include concerns about genetically modified (GM) soyabeans and the high sugar content found in soya milk (Supplementary Table S1). There were no differences in study completion and intervention adherence status (Supplementary Table S3). There were no differences by ethnicity observed across all COM-B domains (data not shown). The physical capability was not identified as a motivator or barrier in this study.

### Adverse events

Among the 118 participants enrolled and randomised into the study, there were a few reported serious adverse events within the supplement arm (Group S) throughout the course of the study such as post-menopausal bleeding, ruptured brain aneurysms and one breast cancer diagnosis^(31)^.

## Discussion

In this study of 118 peri- and post-menopausal Asian women participating in a dietary intervention trial, we found that most women had a positive perception towards soya. Asian women commonly looked for information about soya on the internet, while information from health professionals was least reported. Our findings also show that motivation and opportunity facilitated the regular consumption of soya, while psychological capability was the most common barrier to consumption. Interestingly, women in the soya dietary arm were more likely to report changes in their perception of soya and influence on their consumption over time.

Most women participating in our soya intervention trial were largely motivated to consume soya regularly by automatic and reflective motivation. Participants commonly reported their enjoyment of soya products, regular dietary routine or their belief that soya was good for their health as reasons for consumption (Supplementary Table S1). Previous studies with predominantly Western populations also commonly reported a positive perception towards soya with the most common reasons for consumption being the enjoyment of soya foods and to engage in a more healthy diet^(15,16,20,21,33)^. These findings suggest that personal preferences and the desire to incorporate healthy eating habits are important facilitators to the continued consumption of soya foods.

Our findings also show that social opportunity was an important motivator of consumption. A study by Tu and colleagues^(20)^ observed that French people who lived in Vietnam were more willing to try soya foods as compared to those living in France after assimilating into the local culture. Indeed, research has shown that social norms and the socio-psychological environment play a critical role in determining food choices and eating behaviours^(34,35)^. Within the context of participant adherence, previous dietary intervention trials have shown that social support greatly improved adherence to the intervention^(36,37)^. Therefore, the social opportunity may play a paramount role in facilitating regular consumption in an Asian population setting. Future studies are warranted to confirm the importance of social influences on food choices and regularity of consumption, as well as the impact of this on participation and adherence in dietary intervention trials.

Interestingly, factors within the psychological capability domain were the most commonly reported barriers to consumption, such as knowledge about the type of soyabeans or the content of soya foods. It is likely that the barriers to consuming soya are population dependent. For example, the barriers observed in Western cohort studies were the dislike of the taste, texture or appearance of soya and uncertainty about how to cook or prepare soya^(15,17,19,20)^. Concerns about GM soyabeans were reported only as a minor concern in two studies^(19,20)^. This highlights the importance of ensuring that interventions are acceptable and culturally appropriate to ensure the success of a programme or intervention trial. It also suggests that the barriers to regular soya intake among Asian women may be easily overcome by adequate, accurate information about soya and its effect on health.

Compared to participants in the supplement and negative control arm, soya dietary participants became more motivated to maintain a regular consumption of soya by their intentions and were less likely to report psychological capability as a barrier. Previous studies investigating the effect of intervention in increasing patient physical activity levels reported that engagement with the intervention increases patient knowledge of the health benefits of physical activity and this further improves their ability to maintain activity^(38–40)^. Our results suggest that participants within the dietary soya arm had an improved perception of the health benefits of soya foods over time in a soya intervention trial. In turn, this motivates participants to regularly include soya in their diets. This raises the possibility that understanding perception is a key component to designing effective dietary intervention trials with high adherence. The underlying role of perception in dietary trials should be explored by future studies.

In this cohort, the internet was reported as the most common source of information about soya. Previous studies have emphasised the need for healthcare professionals to play a more active role in communicating clearer messages about soya^(16,18)^. However, the conflicting evidence about the benefits of soya may lead to misinformation for both patients and their healthcare professionals^(15–19)^. This is exemplified in a study by Messina and colleagues^(33)^ which highlighted that Asian healthcare professionals were more likely to believe soya can cause gout, despite the clinical and epidemiological data that have stated otherwise. A stronger evidence base on the health effects of soya is warranted and will promote informed decision-making about incorporating soya into a regular diet.

### Strengths

To our knowledge, this is the first study to describe the perception of soya among women of diverse Asian ancestry living in Malaysia. By comparing women who were randomly assigned to either a dietary, supplement or control arm, we were able to determine whether perception was influenced by their intake of soya. The use of widely acceptable behavioural models, such as the COM-B model and TDF, allows for a strong theoretical basis to characterise the motivators and barriers to regular soya intake in this cohort. This theory-based analysis could be useful to future programmes or studies that are seeking to increase soya intake among Asian populations.

### Limitations

Our cohort was largely comprised of Chinese, highly educated women who are likely to engage in mammography screening. This is likely not reflective of the general population of healthy, peri- or post-menopausal women in Malaysia. Furthermore, it is possible that the women who participated in this study were those who enjoyed soya foods or did not believe it was harmful to their health, which could lead to important biases in the findings. However, our results suggest that some women joined even though they had reservations about the health effects of soya. The findings in our study were limited by the small sample size in terms of comparison of women who were either in the intervention or control arm. While this study was well-powered to detect larger differences in perception between the intervention and control arms, it required a larger sample size to detect smaller differences.

## Conclusion

The findings from this study show that motivation and opportunity are the main drivers of regular soya consumption while knowledge was the most reported barrier to consumption among healthy, Asian post-menopausal women. Using a theory-based approach, we demonstrate that perception is fluid and influenced by knowledge gained over time. We propose that understanding perception within specific cultural and population context could be the solution to improving compliance and retention in dietary intervention trials. Future clinical trials investigating the soya and public health relationship are warranted to enable better communication and informed decision-making between patients and their healthcare professionals.

## Supplementary material

For supplementary material accompanying this paper visit https://doi.org/10.1017/jns.2023.48. click here to view supplementary material
