# Supplementary material for: Perceptions, attitudes and beliefs towards soya among healthy Asian women participating in a soya randomised controlled trial
Source: J Nutr Sci. 2023 Jul 5;12:e69. doi: 10.1017/jns.2023.48 (PMC10345781; doi:10.1017/jns.2023.48)
Supplement: Supplementary file 1 [file S2048679023000484sup001.docx]

Supplementary Table 1: Motivators and barriers as defined by the COM-B model and TDF

| **COM-B Domain** | **TDF Domain** | **Motivator** | **Barrier** | **Perception Survey Statement** |
| --- | --- | --- | --- | --- |
| Psychological Capability | Behavioral regulation |  | - "It's (taking soy daily) a lot […] it's really not something that I look forward to, you know." |  |
|  |  |  | - "Maybe the two servings [of soy] is too much for me.“ |  |
|  |  |  | - "...I just find it (tempeh), for me, really hard to consume." |  |
|  | Knowledge | - "You can have a chance to discuss, to know more, to try something new“ |  | - Soya milk usually has a lot of sugar |
|  |  | - "... good to know that something that I love to eat (soy) can be a prevention of some sort […] something that I knew before but then it’s now for [research]." |  | - I have concerns about genetically modified soybeans |
|  | Skills | - "I was quite excited about it (taking soy), the first two weeks trying recipes“ |  |  |
| Physical Opportunity | Environmental context and resources | - “…soy drink is easy to get because you can just go to the supermarket and you can just buy packets of them." | - “…that’s a hassle looking for soy products and I thought I would just quit, I couldn’t take it anymore.“ | - It is easy to find |
|  |  | - “…I have been like relying on it [soy powder] so much just because it’s the easiest and it’s just there and all I have to do is just warm up the water." | - “[soy milk] is quite expensive.” | - It is cheap |
|  |  |  | - “…if you said that it’s (taking soy everyday) very convenient, then not really..." |  |
| Social Opportunity | Social influences | - "Part of my social support is my family right but a big part of it is also my friends […] so that support was […] quite good and important for me. “ | - "...that lady (food vendor) was telling me, you have been consuming quite a lot [of soy], you know it’s not good for you.“ | - My family likes/does not like soy |
|  |  | - "I think you guys have done you level best you give me support intermittently, that I think is a good reminder that that actually keep one focus as well.“ | - “Well, my cousins had breast cancer [and] they were told to lay off soya products." | - It is part of my culture |
|  |  | - "Maybe if you have like some meetings every couple of months or something to bring people and I don’t know how motivated people are but to maybe to have […] like a competition, a recipe of something." |  | - My family says it is good/harmful for my health |
|  |  |  |  | - My friends say it is good/harmful for my health |
|  |  |  |  | - My doctor says it is good/harmful for my health |
|  |  |  |  | - I heard from media it is good/harmful for health |
|  |  |  |  | - My family does not normally eat soy products |
| Automatic Motivation | Reinforcement | - "...I love soy, so that is a plus factor for me […] and I’m used to [drinking] soy drink.“ |  | - I enjoy eating soy products |
|  |  | - "Plus the fact that you're paying for it (soy products) all." |  | - I don’t like how it tastes |
|  |  |  |  | - I’m not used to it |
| Reflective Motivation | Intentions | - "...his (son's) ex-girlfriend died of cancer [...] in a way I am also just like doing it for her plus if you remember my maternal side has a lot cases[...] of cancer...“ |  | - It is part of my regular diet |
|  |  | - "...when you asked me whether I was willing to participate […] I was like yes! You know, I need to […] help to prevent breast cancer...“ |  |  |
|  |  | - "I feel like I really have to do this (participate in study). Really have to do this. " |  |  |
|  | Beliefs about consequences |  | - “…when you incorporate a lot of tofu in your diet, it’s like you eat tofu but there is a part of the body that also wants to eat chicken...“ | - It is good for my health |
|  |  |  | - "I probably put on weight but I don't know [if] it's because of the soy.“ | - I see no benefit of eating soy |
|  |  |  | - "…initially it was fine until I had diarrhoea." | - I believe it is harmful to my health |
|  |  |  |  | - It makes me gain weight |
|  |  |  |  | - It makes me feel bloated |

Supplementary Table 2: Availability and utilisation of soy products by intervention arm, study completion status and intervention adherence at baseline visit

| **Perception Statement** | **Study Arm (%)** | | |  | **SCS (%)** | |  | **Intervention Adherence (%)** | |  |
| --- | --- | --- | --- | --- | --- | --- | --- | --- | --- | --- |
|  | **S (n = 41)** | **D (n = 38)** | **C (n = 39)** | **p** | **Complete (n = 58)** | **Drop out (n = 21)** | **p** | **Adh**  **(n = 39)** | **Non-adh**  **(n = 19)** | **p** |
| **Where do you normally purchase soy products?** | | | | | | | | | | |
| Wet Market | 10 (24.4) | 15 (39.5) | 21 (53.8) | *0.027 | 20 (34.5) | 5 (23.8) | 0.424 | 15 (38.5) | 5 (26.3) | 0.397 |
| Grocery Stores | 32 (78.0) | 29 (76.3) | 28 (71.8) | 0.831 | 43 (74.1) | 18 (85.7) | 0.370 | 27 (69.2) | 16 (84.2) | 0.340 |
| Restaurant/food stalls | 16 (39.0) | 9 (23.7) | 7 (17.9) | 0.093 | 17 (29.3) | 8 (38.1) | 0.585 | 11 (28.2) | 6 (31.6) | 0.999 |
| I make it myself | 1 (2.4) | 1 (2.6) | 2 (5.1) | 0.842 | 2 (3.4) | 0 (0.0) | 0.999 | 0 (0.0) | 2 (10.5) | 0.103 |
| **How often do you purchase soy products for yourself or your family?** | | | | | | | | | | |
| Daily | 0 (0.0) | 0 (0.0) | 0 (0.0) | 0.231 | 0 (0.0) | 0 (0.0) | 0.649 | 0 (0.0) | 0 (0.0) | 0.931 |
| Few times a week | 11 (26.8) | 14 (36.8) | 6 (15.4) |  | 19 (32.8) | 6 (28.6) |  | 12 (30.8) | 7 (36.8) |  |
| Few times a month | 22 (53.7) | 20 (52.6) | 24 (61.5) |  | 29 (50.0) | 13 (61.9) |  | 20 (51.3) | 9 (47.4) |  |
| Very rarely | 8 (19.5) | 4 (10.5) | 9 (23.1) |  | 10 (17.2) | 2 (9.5) |  | 7 (17.9) | 3 (15.8) |  |
| **On average, how much do you spend on soy products in a month for you and your family?** | | | | | | | | | | |
| RM 10 or less | 9 (22.0) | 9 (23.7) | 6 (15.4) | 0.660 | 14 (24.1) | 4 (19.0) | 0.587 | 9 (23.1) | 5 (26.3) | 0.626 |
| RM10 - 50 | 20 (48.8) | 22 (57.9) | 24 (61.5) |  | 32 (55.2) | 10 (47.6) |  | 22 (56.4) | 10 (52.6) |  |
| RM50 - 100 | 10 (24.4) | 7 (18.4) | 8 (20.5) |  | 11 (19.0) | 6 (28.6) |  | 8 (20.5) | 3 (15.8) |  |
| > RM 100 | 2 (4.9) | 0 (0.0) | 0 (0.0) |  | 1 (1.7) | 1 (4.8) |  | 0 (0.0) | 1 (5.3) |  |
| **What is your main source of information about soy products?** | | | | | | | | | | |
| Family members | 12 (29.3) | 15 (39.5) | 11 (28.2) | 0.499 | 19 (32.8) | 8 (38.1) | 0.789 | 13 (33.3) | 6 (31.6) | 0.999 |
| Friends | 20 (48.8) | 20 (52.6) | 14 (35.9) | 0.301 | 28 (48.3) | 12 (57.1) | 0.612 | 20 (51.3) | 8 (42.1) | 0.583 |
| Newspaper/Radio | 14 (34.1) | 21 (55.3) | 14 (35.9) | 0.117 | 26 (44.8) | 9 (42.9) | 0.999 | 19 (48.7) | 7 (36.8) | 0.417 |
| Internet | 25 (61.0) | 21 (55.3) | 22 (56.4) | 0.893 | 36 (62.1) | 10 (47.6) | 0.306 | 24 (61.5) | 12 (63.2) | 0.999 |
| Public talks | 3 (7.3) | 6 (15.8) | 4 (10.3) | 0.465 | 5 (8.6) | 4 (19.0) | 0.236 | 1 (2.6) | 4 (21.1) | *0.036 |
| Doctors/nurses | 4 (9.8) | 3 (7.9) | 2 (5.1) | 0.835 | 4 (6.9) | 3 (14.3) | 0.375 | 2 (5.1) | 2 (10.5) | 0.591 |
| Others | 2 (4.9) | 3 (7.9) | 6 (15.4) | 0.295 | 3 (5.2) | 2 (9.5) | 0.605 | 1 (2.6) | 2 (10.5) | 0.248 |

Supplementary Table 3: Perception by study completion status and intervention adherence at baseline and end-of-study visit

|  | **Baseline** | | | | | | **End-of-Study** | | | |
| --- | --- | --- | --- | --- | --- | --- | --- | --- | --- | --- |
| **Perception Statement** | **SCS (%)** | | | **Intervention Adherence (%)** | | | **SCS%** | **Intervention Adherence (%)** | | |
|  | **Complete** | **Drop out** | **p** | **Adh** | **Non-adh** | **p** | **Complete (n=58)** | **Adh** | **Non-adh** | **p** |
|  | **(n = 58)** | **(n = 21)** |  | **(n = 39)** | **(n = 19)** |  |  | **(n = 39)** | **(n = 19)** |  |
| **Positive Statement** |  |  |  |  |  |  |  |  |  |  |
| Promotes good health | 50 (86.2) | 15 (71.4) | 0.181 | 35 (89.7) | 15 (78.9) | 0.418 | 51 (87.9) | 36 (92.3) | 15 (78.9) | 0.201 |
| Promotes healthy pregnancy | 22 (37.9) | 7 (33.3) | 0.796 | 14 (35.9) | 8 (42.1) | 0.775 | 14 (24.1) | 12 (30.8) | 2 (10.5) | 0.113 |
| Helps reduce menopause symptoms | 13 (22.4) | 7 (33.3) | 0.384 | 9 (23.1) | 4 (21.1) | 0.999 | 20 (34.5) | 13 (33.3) | 7 (36.8) | 0.999 |
| May reduce risk of BC | 16 (27.6) | 2 (9.5) | 0.131 | 12 (30.8) | 4 (21.1) | 0.541 | 26 (44.8) | 17 (43.6) | 9 (47.4) | 0.999 |
| **Neutral Statement** |  |  |  |  |  |  |  |  |  |  |
| Has no impact on health | 7 (12.1) | 0 (0.0) | 0.18 | 6 (15.4) | 1 (5.3) | 0.408 | 8 (13.8) | 6 (15.4) | 2 (10.5) | 0.999 |
| I have heard other things about soy | 6 (10.3) | 2 (9.5) | 0.999 | 5 (12.8) | 1 (5.3) | 0.653 | 1 (1.7) | 1 (2.6) | 0 (0.0) | 0.999 |
| I have not heard anything about soy and health | 2 (3.4) | 1 (4.8) | 0.999 | 0 (0.0) | 2 (10.5) | 0.103 | 2 (3.4) | 2 (5.1) | 0 (0.0) | 0.999 |
| **Negative Statement** |  |  |  |  |  |  |  |  |  |  |
| Harmful to health | 8 (13.8) | 2 (9.5) | 0.999 | 5 (12.8) | 3 (15.8) | 0.999 | 2 (3.4) | 2 (5.1) | 0 (0.0) | 0.999 |
| Worsens gout | 19 (32.8) | 5 (23.8) | 0.583 | 15 (38.5) | 4 (21.1) | 0.241 | 18 (31.0) | 12 (30.8) | 6 (31.6) | 0.999 |
| Worsens thyroid issues | 1 (1.7) | 0 (0.0) | 0.999 | 1 (2.6) | 0 (0.0) | 0.999 | 3 (5.2) | 1 (2.6) | 2 (10.5) | 0.248 |
| May cause cancer, including BC | 6 (10.3) | 1 (4.8) | 0.668 | 5 (12.8) | 1 (5.3) | 0.653 | 8 (13.8) | 7 (17.9) | 1 (5.3) | 0.252 |
| May trigger cancer, if there is cancer in the family | 3 (5.2) | 0 (0.0) | 0.561 | 1 (2.6) | 2 (10.5) | 0.248 | 2 (3.4) | 2 (5.1) | 0 (0.0) | 0.999 |
| Automatic Motivation | 45 (77.6) | 16 (76.2) | 0.999 | 30 (76.9) | 15 (78.9) | 0.999 | 42 (72.4) | 30 (76.9) | 12 (63.2) | 0.351 |
| Ref. Mot (Belief about Consequences) | 38 (65.5) | 14 (66.7) | 0.999 | 25 (64.1) | 13 (68.4) | 0.999 | 36 (62.1) | 24 (61.5) | 12 (63.2) | 0.999 |
| Ref. Mot (Intentions) | 24 (41.4) | 8 (38.1) | 0.999 | 17 (43.6) | 7 (36.8) | 0.778 | 29 (50.0) | 22 (56.4) | 7 (36.8) | 0.263 |
| Social Opportunity | 42 (72.4) | 12 (57.1) | 0.274 | 29 (74.4) | 13 (68.4) | 0.756 | 39 (67.2) | 28 (71.8) | 11 (57.9) | 0.374 |
| Physical Opportunity | 35 (60.3) | 14 (66.7) | 0.794 | 22 (56.4) | 13 (68.4) | 0.41 | 42 (72.4) | 28 (71.8) | 14 (73.7) | 0.999 |
| Other Reasons | 3 (5.2) | 1 (4.8) | 0.999 | 1 (2.6) | 2 (10.5) | 0.248 | 2 (3.4) | 2 (5.1) | 0 (0.0) | 0.999 |
| Psychological Capability | 20 (34.5) | 5 (23.8) | 0.424 | 13 (33.3) | 7 (36.8) | 0.999 | 8 (13.8) | 5 (12.8) | 3 (15.8) | 0.999 |
| Social Opportunity | 7 (12.1) | 2 (9.5) | 0.999 | 5 (12.8) | 2 (10.5) | 0.999 | 5 (8.6) | 3 (7.7) | 2 (10.5) | 0.999 |
| Reflective Motivation | 6 (10.3) | 2 (9.5) | 0.999 | 5 (12.8) | 1 (5.3) | 0.653 | 5 (8.6) | 1 (2.6) | 4 (21.1) | *0.036 |
| Automatic Motivation | 3 (5.2) | 0 (0.0) | 0.561 | 1 (2.6) | 2 (10.5) | 0.248 | 1 (1.7) | 1 (2.6) | 0 (0.0) | 0.999 |
| Physical Opportunity | 2 (3.4) | 0 (0.0) | 0.999 | 1 (2.6) | 1 (5.3) | 0.999 | 1 (1.7) | 1 (2.6) | 0 (0.0) | 0.999 |
| Other Reasons | 3 (5.2) | 0 (0.0) | 0.561 | 2 (5.1) | 1 (5.3) | 0.999 | 4 (6.9) | 2 (5.1) | 2 (10.5) | 0.591 |

SCS, study completion status; p, p-value calculated using a Fisher’s Exact test; Adh, adherence; Non-adh, non-adherence; BC, breast cancer; Ref. Mot, Reflective Motivation. *P<0.05

Supplementary Table 4: Perception of health effects of soy, motivators and barriers to the regular consumption of soy by study arm, study completion status and intervention adherence status at three-month visit.

| **Perception Statement** | **Study Arm (%)** | | | **SCS (%)** | | | **Intervention Adherence (%)** | | |
| --- | --- | --- | --- | --- | --- | --- | --- | --- | --- |
|  | **S (n = 36)** | **D (n =34)** | **p** | **Complete (n = 57)** | **Drop-out (n = 13)** | **p** | **Adh (n = 39)** | **Non-adh (n = 18)** | **p** |
|  |  |  |  |  |  |  |  |  |  |
| **Positive Statement** | | | | | | | | | |
| Promotes good health | 29 (80.6) | 30 (88.2) | 0.515 | 48 (84.2) | 11 (84.6) | 0.999 | 34 (87.2) | 14 (77.8) | 0.443 |
| Promotes healthy pregnancy | 15 (41.7) | 13 (38.2) | 0.811 | 48 (84.2) | 11 (84.6) | 0.999 | 16 (41.0) | 5 (27.8) | 0.389 |
| Helps reduce menopause symptoms | 10 (27.8) | 9 (26.5) | 0.999 | 15 (26.3) | 4 (30.8) | 0.739 | 11 (28.2) | 4 (22.2) | 0.753 |
| May reduce risk of BC | 13 (36.1) | 11 (32.4) | 0.804 | 19 (33.3) | 5 (38.5) | 0.753 | 13 (33.3) | 6 (33.3) | 0.999 |
| **Neutral Statement** | | | | | | | | | |
| Has no impact on health | 2 (5.6) | 2 (5.9) | 0.999 | 4 (7.0) | 0 (0.0) | 0.999 | 4 (10.3) | 0 (0.0) | 0.297 |
| I have heard other things about soy | 3 (8.3) | 0 (0.0) | 0.24 | 3 (5.3) | 0 (0.0) | 0.999 | 3 (7.7) | 0 (0.0) | 0.544 |
| I have not heard anything about soy and health | 2 (5.6) | 0 (0.0) | 0.493 | 1 (1.8) | 1 (7.7) | 0.339 | 0 (0.0) | 1 (5.6) | 0.316 |
| **Negative** **Statement** | | | | | | | | | |
| Harmful to health | 1 (2.8) | 4 (11.8) | 0.192 | 4 (7.0) | 1 (7.7) | 0.999 | 4 (10.3) | 0 (0.0) | 0.297 |
| Worsens gout | 8 (22.2) | 10 (29.4) | 0.588 | 13 (22.8) | 5 (38.5) | 0.296 | 11 (28.2) | 2 (11.1) | 0.191 |
| Worsens thyroid issues | 0 (0.0) | 3 (8.8) | 0.109 | 3 (5.3) | 0 (0.0) | 0.999 | 3 (7.7) | 0 (0.0) | 0.544 |
| May cause cancer, including BC | 0 (0.0) | 5 (14.7) | *0.023 | 5 (8.8) | 0 (0.0) | 0.576 | 5 (12.8) | 0 (0.0) | 0.168 |
| May trigger cancer, if there is cancer in the family | 0 (0.0) | 2 (5.9) | 0.232 | 2 (3.5) | 0 (0.0) | 0.999 | 2 (5.1) | 0 (0.0) | 0.999 |
| **Motivators** | | | | | | | | | |
| Automatic Motivation | 24 (66.7) | 27 (79.4) | 0.287 | 40 (70.2) | 11 (84.6) | 0.491 | 27 (69.2) | 13 (72.2) | 0.999 |
| Ref. Mot (Belief about Consequences) | 25 (69.4) | 24 (70.6) | 0.999 | 39 (68.4) | 10 (76.9) | 0.741 | 26 (66.7) | 13 (72.2) | 0.766 |
| Ref. Mot (Intentions) | 14 (38.9) | 17 (50.0) | 0.471 | 25 (43.9) | 6 (46.2) | 0.999 | 16 (41.0) | 9 (50.0) | 0.575 |
| Social Opportunity | 23 (63.9) | 22 (64.7) | 0.999 | 37 (64.9) | 8 (61.5) | 0.999 | 26 (66.7) | 11 (61.1) | 0.769 |
| Physical Opportunity | 22 (61.1) | 22 (64.7) | 0.808 | 34 (59.6) | 10 (76.9) | 0.345 | 24 (61.5) | 10 (55.6) | 0.774 |
| Other Reasons | 1 (2.8) | 2 (5.9) | 0.609 | 2 (3.5) | 1 (7.7) | 0.466 | 2 (5.1) | 0 (0.0) | 0.999 |
| **Barriers** | | | | | | | | | |
| Psychological Capability | 6 (16.7) | 9 (26.5) | 0.389 | 12 (21.1) | 3 (23.1) | 0.999 | 9 (23.1) | 3 (16.7) | 0.734 |
| Social Opportunity | 4 (11.1) | 2 (5.9) | 0.674 | 5 (8.8) | 1 (7.7) | 0.999 | 4 (10.3) | 1 (5.6) | 0.999 |
| Reflective Motivation | 2 (5.6) | 5 (14.7) | 0.253 | 2 (3.5) | 5 (38.5) | *0.002 | 1 (2.6) | 1 (5.6) | 0.536 |
| Automatic Motivation | 0 (0.0) | 1 (2.9) | 0.486 | 1 (1.8) | 0 (0.0) | 0.999 | 1 (2.6) | 0 (0.0) | 0.999 |
| Physical Opportunity | 1 (2.8) | 2 (5.9) | 0.609 | 2 (3.5) | 1 (7.7) | 0.466 | 1 (2.6) | 1 (5.6) | 0.536 |
| Other Reasons | 4 (11.1) | 2 (5.9) | 0.674 | 3 (5.3) | 3 (23.1) | 0.073 | 2 (5.1) | 1 (5.6) | 0.999 |

SCS, study completion status; S, supplement arm; D; dietary arm; C, control arm; p, p-value calculated using a Fisher’s Exact test; Adh, adherence; Non-adh, non-adherence; BC, breast cancer; Ref. Mot, Reflective Motivation. *P<0.05

Supplementary Table 5: Statistical power to detect a change in prevalence from the control to intervention arm, assuming a sample size of 30 per arm and Type I error (alpha) of 0.05.

| Prevalence in Control Arm (%) |  | Prevalence in Intervention Arm (%) | | | | | | | |
| --- | --- | --- | --- | --- | --- | --- | --- | --- | --- |
|  | 5% | 10% | 20% | 30% | 40% | 50% | 60% | 70% | 80% |
| 5% | - | 12% | 45% | 78% | 94% | 99% | 100% | 100% | 100% |
| 10% | 12% | - | 20% | 52% | 80% | 95% | 99% | 100% | 100% |
| 20% | 45% | 20% | - | 15% | 40% | 70% | 91% | 98% | 100% |
| 30% | 78% | 52% | 15% | - | 13% | 36% | 66% | 89% | 98% |
| 40% | 94% | 80% | 40% | 13% | - | 12% | 34% | 66% | 91% |
| 50% | 99% | 95% | 70% | 36% | 12% | - | 12% | 36% | 70% |
| 60% | 100% | 99% | 91% | 66% | 34% | 12% | - | 13% | 40% |
| 70% | 100% | 100% | 98% | 89% | 66% | 36% | 13% | - | 15% |
| 80% | 100% | 100% | 100% | 100% | 91% | 70% | 40% | 15% | - |
